# Safety and efficacy of remote ischemic conditioning in adult moyamoya disease patients undergoing revascularization surgery: a pilot study

**DOI:** 10.3389/fneur.2023.1200534

**Published:** 2023-07-28

**Authors:** Heng Yang, Zhenzhen Hu, Xinjie Gao, Jiabin Su, Hanqiang Jiang, Shaoxuan Yang, Qing Zhang, Wei Ni, Yuxiang Gu

**Affiliations:** ^1^Department of Neurosurgery, Huashan Hospital, Fudan University, Shanghai, China; ^2^Neurosurgical Institute, Fudan University, Shanghai, China; ^3^Shanghai Clinical Medical Center of Neurosurgery, Shanghai, China; ^4^National Center for Neurological Disorders, Huashan Hospital, Shanghai Medical College, Fudan University, Shanghai, China; ^5^Department of Neurosurgery, Huashan Hospital North, Fudan University, Shanghai, China; ^6^Department of Nursing, Huashan Hospital North, Fudan University, Shanghai, China

**Keywords:** moyamoya disease, remote ischemic conditioning, complications, modified Rankin Scale (mRS), revascularization surgery

## Abstract

**Background and purpose:**

Revascularization surgery for patients with moyamoya disease (MMD) is very complicated and has a high rate of postoperative complications. This pilot study aimed to prove the safety and efficacy of remote ischemic conditioning (RIC) in adult MMD patients undergoing revascularization surgery.

**Methods:**

A total of 44 patients with MMD were enrolled in this single-center, open-label, prospective, parallel randomized study, including 22 patients assigned to the sham group and 22 patients assigned to the RIC group. The primary outcome was the incidence of major neurologic complications during the perioperative period. Secondary outcomes were the modified Rankin Scale (mRS) score at discharge, at 90 days post-operation, and at 1 year after the operation. The outcome of safety was the incidence of adverse events associated with RIC. Blood samples were obtained to monitor the serum concentrations of cytokines (VEGF, IL-6).

**Results:**

No subjects experienced adverse events during RIC intervention, and all patients could tolerate the RIC intervention in the perioperative period. The incidence of major neurologic complications was significantly lower in the RIC group compared with the control group (18.2% vs. 54.5%, *P* = 0.027). The mRS score at discharge in the RIC group was also lower than the control group (0.86 ± 0.99 vs. 1.18 ± 1.22, *P* = 0.035). In addition, the serum IL-6 level increased significantly at 7 days after bypass surgery in the control group and the serum level of VEGF at 7 days post-operation in the RIC group.

**Conclusion:**

In conclusion, our study demonstrated the neuroprotective effect of RIC by reducing perioperative complications and improving cerebral blood flow in adult MMD patients undergoing revascularization surgery. Thus, RIC seems to be a potential treatment method for MMD.

**Clinical trial registration:**

ClinicalTrials.gov, identifier: NCT05860946.

## Introduction

Moyamoya disease (MMD) is a progressive disease of the proximal cerebral blood vessels. It is characterized by the narrowing of the internal carotid artery, middle cerebral artery, and anterior cerebral artery, and the formation of fragile collateral blood vessels such as a puff of smoke, which resulted in recurrent ischemic stroke or intracranial hemorrhage. Nowadays, extracranial–intracranial revascularization was considered to be standard therapy for MMD patients, including direct, indirect, and combined revascularization, which could decrease the incidence rate of stroke (1–3). Although surgical revascularization could increase cerebral blood perfusion, there are still several postoperative complications, such as ischemic infarction, hemorrhage, and hyperperfusion injury, which would attenuate the therapeutic effects of revascularization. Therefore, how to decrease perioperative complications was the major factor for clinical outcomes in adult MMD patients treated with surgical revascularization.

Remote ischemic conditioning (RIC) is a non-invasive therapeutic approach for protecting organs or tissue against the detrimental effects of acute ischemia-reperfusion injury. Many protective factors produced by the stimulus of RIC could protect remote target organs and tissues by inhibiting oxidation and inflammation. The phenomenon of this protection effect was first found in myocardium ischemia-reperfusion injury (4), and then, RIC was used in children's cardiac surgery to provide myocardial protection during operation (5). RIC was gradually applied to brain protection, and a series of clinical researches have confirmed that it could improve cerebral perfusion status, increase cerebral tolerance to ischemic injury, reduce perihematomal edema, and promote clearance (6). Recently, a randomized controlled study reported that daily RIC could improve cerebral perfusion and slow arterial progression of adult MMD (7). Meanwhile, a single-arm open-label study also indicated that RIC was a promising non-invasive method for ischemic MMD control by relieving symptoms and reducing stroke recurrence (8). In addition, the effects of RIC on reducing neurologic complications in MMD patients treated with revascularization surgery have also been reported (9, 10). However, the mechanism of RIC in reducing perioperative complications for MMD patients is still unknown. Thus, we conducted a randomized controlled study to explore the safety and efficacy of RIC in adult MMD patients undergoing revascularization therapy.

## Methods

### Study design

**Figure 1:**
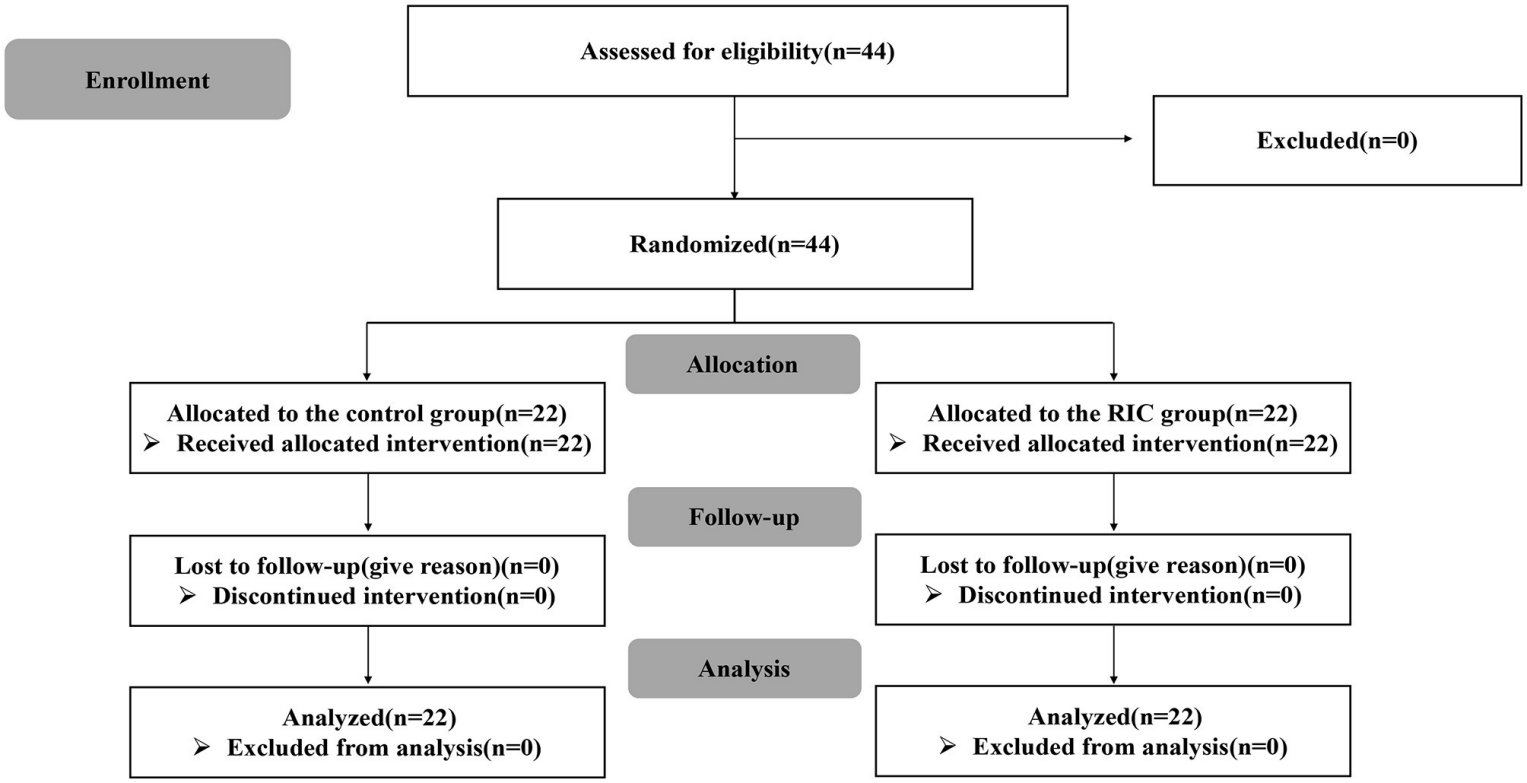
Flow chart of patient enrollment.

This was a single-center, open-label, prospective, parallel randomized study from April 2020 to April 2021 in adult MMD patients at Huashan Hospital, Fudan University. This study was registered at ClinicalTrials.gov with the unique identifier NCT05860946. To determine whether RIC could provide neuroprotective effects for adult MMD patients treatment with surgical revascularization, subjects were randomly assigned in a 1:1 ratio to the RIC group and the control group and followed for 3 and 12 months after revascularization, respectively. The flow chart of patient enrollment is shown in Figure 1. This study was approved by the Ethics Committee of Huashan Hospital, Fudan University (Approval number: 2019329). All subjects or their legally authorized representatives provided informed consent before enrollment.

### Interventions

Patients in the RIC and control group achieved standard sub-temporal artery (STA) to middle cerebral artery (MCA) bypass and encephaloduromyosynangiosis (EDMS). All surgeries were performed by experienced neurosurgeons who have performed over 100 revascularization surgeries. Patients in the RIC group and control group will achieve RIC intervention and sham RIC intervention three times daily from 5 days before the operation and 7 days post-operation. The RIC intervention included five cycles of 5 min inflating tourniquets with a pressure of 200 mm Hg and 5 min deflating with a pressure of 0 mm Hg alternately. Patients in the control group, with bilateral upper arm cuffs, were inflated to a pressure of 60 mm Hg for 5 min, followed by 5 min of relaxation of the cuffs. RIC and sham RIC were performed in the hospital under the supervision of an investigator. The RIC process would be stopped at any time if the subject experienced any discomfort.

### Subject population

Subjects were eligible for enrollment if they met all of the inclusion criteria:

1. Patients aged from 18 to 65 years old.
2. Subjects all performed digital subtraction angiography (DSA) and were diagnosed with MMD according to the criteria recommended by the Research Committee on MMD (Spontaneous Occlusion of the Circle of Wills) of the Ministry of Health and Welfare of Japan in 2012 (11).
3. Modified Rankin Scale (mRS) score < 4.
4. Informed consent is obtained from the patient or legally authorized representative.

Subjects who met any of the following exclusion criteria were excluded from this study:

1. Subjects who suffer from acute ischemic or hemorrhagic stroke within 3 months.
2. Severe hepatic or renal dysfunction.
3. Severe cardiac disease.
4. Severe hemostatic disorder or severe coagulation dysfunction.
5. Serious, advanced, or terminal illnesses with an anticipated life expectancy of < 1 year.
6. Patients with moyamoya syndrome caused by autoimmune disease, Down's syndrome, neurofibromatosis, leptospiral infection, or previous skull-base radiation therapy.

### Randomization and masking

The subjects diagnosed with MMD by DSA who had not been accepted for revascularization surgery were recruited. After baseline assessment, eligible patients signed informed consent. They were randomized in a 1:1 ratio to accept either RIC plus routine medical treatment or routine medical treatment only with a computer-generated randomization code. The code was put into an opaque envelope. The investigators would number the eligible patients and open the envelope to determine the treatment plan. Investigators who assessed outcomes were blinded to the allocation.

### Outcome assessment

The primary outcome was the incidence of major neurologic complications during the perioperative period, such as hyperperfusion syndrome, cerebral infarction, intracranial hemorrhage, and seizure. Secondary outcomes were the modified Rankin Scale (mRS) score at discharge, at 90 days post-operation, and at 1 year after the operation. The outcome of safety was the incidence of adverse events associated with RIC. A neurosurgeon was blinded to the group assignment and evaluated the major neurologic complications based on clinical symptoms and radiologic images. Neuroradiologic outcomes were assessed by two experienced radiologists who were blinded to the allocation. Patients performed PCASL-MR before the operation and at 7 days after the operation to assess the cerebral perfusion. In addition, the revascularization evaluation after bypass surgery was assessed by digital subtraction angiography (DSA) which was performed at 6 months after the operation. Postoperative angiographic outcomes are commonly assessed according to Matsushima grade, categorized into A (more than two-thirds of the MCA territory supplied by the bypass), B (one-third to two-thirds), and C (less than one-third) (12).

### Blood samples and cytokine analysis

Blood samples were drawn from the cubital vein; the points of measurement included baseline, post-RIC (before operation), and post-RIC (7 days post-operation). Serum levels of VEGF (ab100662) and IL-6 (ab178013) were evaluated using the enzyme-linked immunosorbent assay (ELISA), following the manufacturer's instructions and as described previously (13). A standard curve was built for each set of samples, and the biomarkers were assayed, yielding a correlation coefficient in the range of 0.98 to 0.99. It is worth mentioning that the serum concentrations of cytokines (VEGF, IL-6) were normalized using the total protein concentration (Thermo Scientific Pierce™ BCA Protein Assay Kit, MA, USA).

### Statistical analysis

To compare the characteristics at the baseline or follow-up of the RIC group with the control group, continuous variables described as mean ± SD, or median (IQRs) were analyzed by the independent Student's *t*-test or the Mann–Whitney *U*-test; categorical variables described as proportions were analyzed by the chi-square test or the Fisher exact test using SPSS Statistics version 23 (IBM Incorporation, Armonk, New York, USA). To further evaluate the effect of RIC on the progression of stenotic-occlusive lesions, the binary logistic regression analysis was used to determine the odds ratio (OR) with RIC. For comparing the cumulative incidence of MACEs at 12 months, the Cox proportional hazards models were used. Interrater agreements of CBF measurements were evaluated by intraclass correlation coefficients (ICC). Consistency between two radiologists is considered good when the ICC value is ≥ 0.75. A *p*-value of < 0.05 was considered to be statistically significant for all the tests. All the tests were two-sided.

## Results

### Baseline characteristics

Between April 2020 and April 2021, we enrolled 44 MMD patients undergoing revascularization surgery, including 22 patients assigned to the sham group and 22 patients assigned to the remote ischemic conditioning group. The average ages were 43.9 ± 9.81 and 44.4 ± 8.86, respectively, in the control group and the RIC group. There were 17 patients who presented with TIA, 17 patients with ischemic stroke, and the remaining patients presented with hemorrhagic stroke. Almost all patients achieved combined revascularization; moreover, one patient performed indirect revascularization. The baseline characteristics of 44 subjects are summarized in Table 1.

**Table 1:** Baseline characteristics of MMD patients.

**Characteristics**	**Control group**	**RIC group**	***P*-value**
Female (%)	9 (40.9)	12 (52.2)	0.547
Age	43.9 ± 9.81	44.4 ± 8.86	0.64
Suzuki stage	3.95 ± 0.99	3.68 ± 0.94	0.8
Lesion for revascularization			0.76
Left side	14	12	
Right side	8	10	
Preoperative symptoms			0.74
TIA	10	7	
Ischemic stroke	8	9	
Hemorrhagic stroke	4	6	
Surgical methods			1
Indirect revascularization	1	0	
Combine revascularization	21	22	
Hypertension (%)	8 (36%)	7 (32%)	1
Diabetes (%)	3 (14%)	4 (18%)	1
Drink (%)	8 (36%)	7 (32%)	1
Smoke (%)	10 (45%)	8 (36%)	0.76
Operation time (mins)	146.5 ± 2.763	144.1 ± 2.273	0.37

### Safety and efficacy outcomes

**Figure 2:**
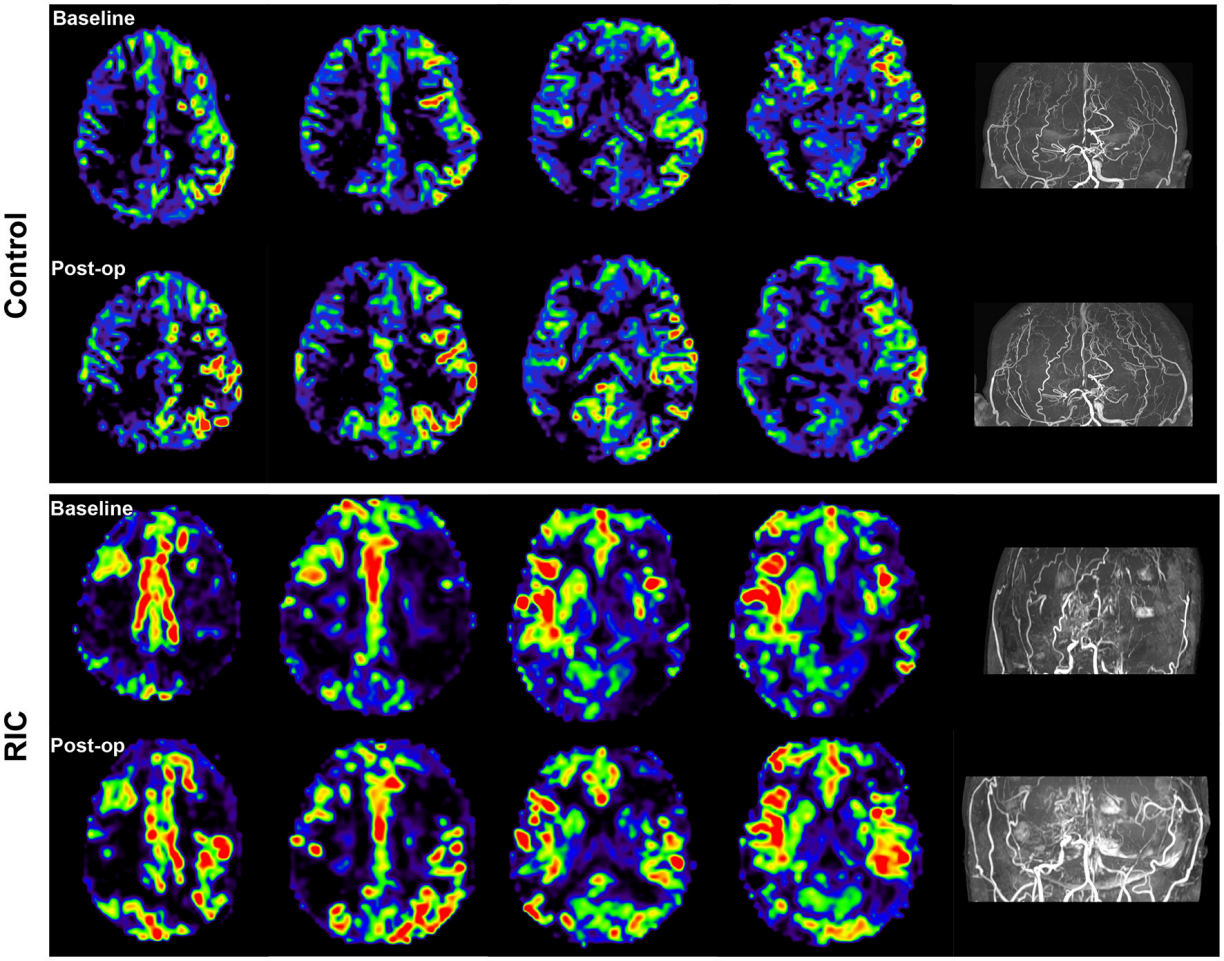
Cerebral blood flow at the baseline and 7 days after operation. Arterial spin labeling (ASL) images of the patient in both the control and RIC groups demonstrated remarkable perfusion improvement after bypass surgery, especially in the RIC group.

No subjects experienced adverse events during RIC intervention, and all patients can tolerate the RIC intervention in the perioperative period. Major neurologic complications occurred in 54.5% of patients in the control group, including three patients suffering from ischemic infarction, seven patients presented hyperperfusion syndrome, and two patients presented seizure attacks. In the RIC group, the incidence of major neurologic complications was 18.2%, one patient suffered ischemic infarction, two patients suffered hyperperfusion syndrome, and one patient suffered a seizure attack. Obviously, the incidence of major neurologic complications was significantly lower in the RIC group compared with the control group (*P* = 0.027). In addition, the mRS score at discharge in the RIC group was also lower than in the control group (0.86 ± 0.99 vs. 1.18 ± 1.22, *P* = 0.035). ASL images of patients evaluated at both the baseline and 7 days post-operation demonstrated remarkable perfusion improvement after bypass surgery, especially in the RIC group (Figure 2). However, according to the DSA at 6 months after bypass surgery, the result of Matsushima grade showed that there was no evidence of a significant difference in postoperative angiographic outcomes between the control group and the RIC group (Table 2).

**Table 2:** Neurologic outcome in MMD patients between Control and RIC group.

**Variable**	**Control group**	**RIC group**	***P*-value**
Postoperative complications	12	4	0.027
Postoperative infarction	3	1	0.3
Postoperative hemorrhage	0	0	NA
Hyperperfusion syndrome	7	2	0.06
Seziure	2	1	0.55
Matsushima grade			0.83
A	6	8	
B	13	12	
C	3	2	
mRS after operation	1.18 ± 1.22	0.86 ± 0.99	0.035
mRS 90 days post-op	0.68 ± 0.83	0.45 ± 0.67	0.43
mRS 1-year post-op	0.59 ± 0.85	0.31 ± 0.57	0.42
Recurrent stroke within 1 year after operation	2	1	0.53

### Serum cytokine results

**Figure 3:**
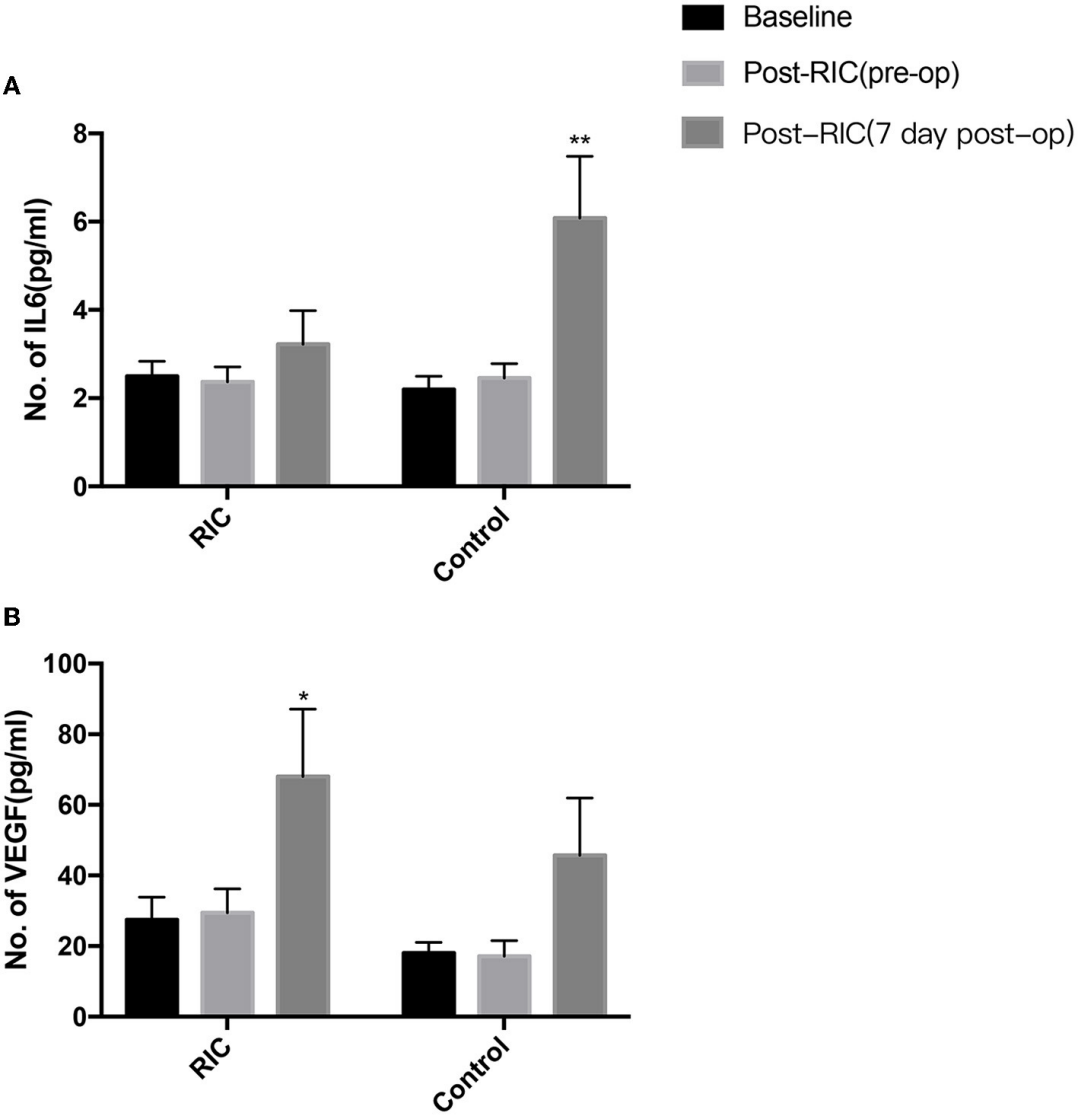
Changes in serum cytokines for MMD patients undergoing revascularization surgery after RIC. The serum IL-6 level increased significantly at 7 days after revascularization surgery in the control group but was not significantly different between pre- and post-operation in the RIC group **(A)**. RIC could also significantly elevate the level of VEGF at 7 days post-operation in the RIC group, but the change of the VEGF level had no obvious difference after operation in the control group **(B)** (*P < 0.05, **P < 0.01 vs. baseline).

Figure 3 shows the evaluation of the vascular endothelial growth factor (VEGF) and IL-6 pre- and post-RIC in both the RIC group and the control group. The serum IL-6 level increased significantly at 7 days after bypass surgery in the control group but was not significantly different between pre- and post-operation in the RIC group. However, RIC could significantly elevate the level of VEGF at 7 days post-operation.

## Discussion

This randomized clinical trial showed that it was safe to perform RIC intervention in MMD patients undergoing bypass surgery, and RIC could decrease the incidence of post-operation complications significantly but did not significantly promote the clinical outcomes at 1 year after the operation and reduce the occurrence of strokes within 1 year after bypass surgery.

Currently, revascularization surgery has been established as an effective treatment for MMD, including direct or indirect revascularization. Recent studies reported that revascularization surgery for adult patients with ischemic MMD could improve both cerebral perfusion of middle cerebral artery territory and cognitive function (14, 15). Other studies also indicated that revascularization surgery could also reduce the incidence of rebleeding for hemorrhagic MMD patients and improve long-term outcomes (3, 16). However, there was still a high rate of perioperative complications of bypass surgery for MMD patients, such as ischemic stroke, cerebral hemorrhage, hyperperfusion syndrome, and seizure attack. Unfortunately, there is no effective way to avoid perioperative complications of bypass surgery. In our study, we found that RIC could decrease the incidence of post-operation complications significantly. Therefore, RIC intervention may be a potential effective treatment for MMD.

Remote ischemic conditioning is a therapeutic strategy for the protection of tissue/organs against the detrimental effects of ischemia-reperfusion injury. The protection effect of RIC was first described in 1986 by Murry et al. (4) in the setting of experimental myocardial infarction. Later, its beneficial effects are also seen in other organs (the lungs, liver, kidney, intestine, and brain) subject to acute ischemia-reperfusion injury (17). Nowadays, RIC has been established as a potentially powerful therapeutic tool for many diseases. Multiple previous studies have evaluated the effect of RIC on neuroprotection, and RIC intervention has been suggested to treat acute ischemic stroke and intracranial arterial stenosis (6, 18–20). In addition, it is also effective of RIC in reducing the incidence of postoperative ischemic stroke in patients who underwent brain tumor surgery and patients with subarachnoid hemorrhage (21, 22). Recently, RIC has been applied to the treatment of MMD, which could not only improve cerebral perfusion and relieve symptoms but also reduce neurologic complications for MMD patients undergoing revascularization surgery therapy (8, 9). A previous study has also reported that RIC could slow the arterial progression of the stenotic-occlusive lesions in patients with MMD (7) and reduce the incidence of recurrent stroke (8). However, the protective effect of RIC was only effective in the perioperative period, and RIC was ineffective in promoting the clinical outcomes at 1 year after the operation and reducing the recurrence of strokes within 1 year after bypass surgery in our study, which may be due to a short period of RIC intervention. Two previous trials showed that a longer period (180 days or 300 days) of RIC exercise could prevent recurrent ischemic stroke (23, 24). Therefore, it is necessary for a long-period RIC intervention to improve clinical outcomes and reduce the rate of recurrent stroke for MMD patients. However, the optimal dose and intervention protocol for RIC intervention are still unknown and need further investigation.

The mechanism of the protective effect of RIC in MMD is not clear and still needs further study. Currently, the potential mechanisms of RIC on neuroprotection include anti-inflammatory response, reduced excitotoxicity, neurovascular protection, and metabolic protection (25, 26). RIC plays a role in affecting critical proteins and regulating gene regulation and new protein synthesis (27). There are three routes (humoral, neuronal, and immunologic pathways) of protective signals that may be transmitted from the conditioned organ to the brain (28), and those three pathways interacted with each other and are not necessarily mutually exclusive. For MMD, the potential mechanisms of RIC on neuroprotection might be reducing hyperperfusion syndrome and promoting the recovery of blood flow after revascularization therapy. RIC intervention could improve nitric oxide (NO) production, which could increase cerebral blood flow, improve microvascular perfusion, and maintain brain hemostasis (22, 29). In our study, RIC intervention could significantly elevate the level of VEGF after bypass surgery, which could enhance angiogenesis and increase cerebral circulation. Thus, the cerebral blood flow measured by ASL in patients of the RIC group after bypass surgery was increased obviously compared with the control group. As we all know, inflammation plays an important role in developing cerebral hyperperfusion injury after bypass surgery in patients with MMD. We found that the serum IL-6 level increased significantly at 7 days after bypass surgery, but RIC intervention could decrease the serum level of IL-6. Therefore, RIC could suppress the neuroinflammatory response after revascularization by lowering serum inflammatory cytokines.

Our study has some limitations. First, the sample size was relatively small and may bring selection bias for this study. The second limitation of this study was that the period of RIC intervention was too short to achieve a long-term good outcome in patients undergoing bypass surgery. Numerous previous studies have suggested that a long period of RIC intervention could improve clinical outcomes and reduce the rate of recurrent stroke for MMD patients. Moreover, we just collect blood samples at three time points to observe the changes in serum cytokines expression before and after RIC due to the difficulties of obtaining the consent of participants and approval of the ethics committee. Finally, the exclusion criteria were unrigorous: patients with severe existing neurologic or psychiatric disease, patients with a history of revascularization surgery, and patients with cancer should be also excluded from this study.

## Conclusion

Our study demonstrated the neuroprotective effect of RIC by reducing perioperative complications and improving cerebral blood flow in adult MMD patients undergoing revascularization surgery. Thus, RIC seems to be a potential treatment method for MMD.

## Data availability statement

The original contributions presented in the study are included in the article/supplementary material, further inquiries can be directed to the corresponding authors.

## Ethics statement

The studies involving human participants were reviewed and approved by the Huashan Institutional Review Board. The patients/participants provided their written informed consent to participate in this study. Written informed consent was obtained from the individual (s) for the publication of any potentially identifiable images or data included in this article.

## Author contributions

HY and ZH designed the research and drew graphs. HY, ZH, XG, JS, and HJ performed research. QZ, XG, and SY analyzed data. HY, ZH, YG, and WN wrote the article. All authors read and approved the final manuscript.
